# Impacts of noise-induced hearing loss on sleep, health, and workplace: Multi-group analysis

**DOI:** 10.1016/j.heliyon.2024.e30861

**Published:** 2024-05-08

**Authors:** Hyeon Jo, Eun-Mi Baek

**Affiliations:** a Headquarters, HJ Institute of Technology and Management, 71 Jungdong-ro 39, Bucheon-si, Gyeonggi-do, 14721, Republic of Korea; b Department of Preventive Medicine, College of Medicine, Catholic University of Korea, 222 Banpo-daero, Seocho-gu, Seoul, 06591, Republic of Korea

**Keywords:** NIHL, Occupational health, Sleep quality, Daily life health conditions, Workplace health problems

## Abstract

Noise-induced hearing loss (NIHL) is a significant occupational health concern, particularly in industries with high levels of noise exposure. This study examines the effects of NIHL on sleep quality, daily life health conditions, and workplace health problems among workers. A total of 1285 workers participated in the study, and the data were analyzed using partial least squares structural equation modeling (PLS-SEM) to assess the impacts of NIHL. The analysis included a multi-group analysis to differentiate the effects between workers who wear noise protection and those who do not. Our findings indicate that NIHL significantly affects sleep quality, with a coefficient of 0.263 (*t* = 9.957, *p*<0.001), daily life health conditions with a coefficient of 0.296 (*t* = 10.793, *p*<0.001), and workplace health problems with a coefficient of 0.345 (*t* = 13.814, *p*<0.001). The multi-group analysis revealed more severe impacts on sleep and health in the non-wearing group compared to the noise-protection-wearing group, with statistically significant differences in path coefficients for sleep disorders (−0.033), health problems in daily life (−0.184), and health problems in the workplace (−0.190), all showing *p-*values of 0.000. These results underscore the detrimental effects of NIHL on multiple aspects of workers' health and emphasize the importance of wearing noise protection to mitigate these effects. This study provides vital insights for both researchers and practitioners in public health, suggesting that improved noise protection strategies are essential for protecting workers in noisy environments.

## 1 Introduction

Noise-induced hearing loss (NIHL) is an extensive global health concern affecting over 500 million people worldwide, with 25 % of workers exposed to hazardous noise levels [1,2]. NIHL is a permanent hearing impairment resulting from prolonged exposure to high levels of noise [3]. This type of hearing loss is characterized by a gradual, cumulative, and often irreversible decline in auditory function. NIHL typically occurs after years of exposure but can also result from a single exposure to intense noise, such as an explosion. It primarily affects the frequency ranges vital for understanding speech, thereby significantly impairing communication ability [4,5]. Common sources of noise leading to NIHL include industrial machinery, loud music, and transportation. Prevention strategies involve using protective devices, reducing noise exposure, and regular auditory check-ups [6,7]. These exposures are not only linked to auditory issues but also to a wide range of psychological and cardiovascular problems, including tinnitus, depression, hypertension, and sleep disturbances, impacting a significant number of the workforce [8,9]. Research indicates a robust connection between NIHL and sleep disruptions, suggesting that continuous noise exposure, whether from industrial activities or environmental sources like wind turbines, severely affects sleep quality. For instance, a study at the Manjil wind farm in northern Iran revealed that wind turbine noise substantially disturbs sleep patterns among workers, manifesting in increased daytime sleepiness and degraded sleep quality [10]. Similar findings were observed in the textile industry, where noise levels significantly impacted workers' sleep, confirming that environmental noise is a critical determinant of sleep health [11]. Furthermore, NIHL extends its impact to job stress and overall workplace well-being. A structural equation model in the textile industry illustrated how noise exposure exacerbates job stress, mediated by job satisfaction and noise sensitivity, underscoring the complex interplay between workplace noise and worker stress [12]. This exposure also contributes to broader health issues such as elevated blood pressure and cardiovascular risks, facilitated by stress responses that activate biological risk factors through the autonomic nervous and endocrine systems [9,13]. Additionally, hospital environments reveal that noise not only annoys but significantly compromises the quality of patient care. High noise levels negatively affect staff's cognitive function and emotional state, which in turn, impacts patient safety and care quality, mediated by noise sensitivity and annoyance among healthcare providers [14]. The potential role of noise exposure in increasing cancer risk has also been explored in recent studies. A comprehensive systematic review associated prolonged noise exposure with specific types of cancer, such as acoustic neuroma and breast cancer, suggesting that long-term exposure to high noise levels could be a contributing factor to cancer incidence [15]. The wide-ranging effects of NIHL highlight the critical need for rigorous noise control measures and personal protective strategies to mitigate these risks in occupational settings and beyond. These interventions are essential not only for protecting hearing but also for preserving overall health and well-being in noise-exposed populations.

To prevent NIHL and its associated health issues, it is crucial to minimize the health effects of noise exposure, provide workers with hearing protection, and establish safe working environments. Research by Wang, Chang [16] suggests that workplace interventions, such as hearing protection programs, can reduce the incidence of cardiovascular disease.

This research paper addresses gaps in the existing literature concerning NIHL. Previous studies primarily focused on the audiological aspects of NIHL, yet comprehensive research investigating its effects on sleep quality and daily health conditions is lacking. This study aims to fill this void by examining the specific impacts of NIHL on sleep disturbances, including sleep duration, sleep efficiency, and sleep disorders. Additionally, it will assess the broader health implications of NIHL, including its association with physical and mental health problems such as cardiovascular issues, stress-related disorders, and psychological well-being. Despite the crucial role of hearing protection in preventing NIHL, research on the differential impacts of NIHL based on the usage of noise protection is limited. This study addresses this gap through a multi-group analysis (MGA) comparing outcomes between workers who wear noise protection and those who do not. The main objectives of this research paper are: 1) To examine the impacts of NIHL on sleep quality among workers, 2) To assess the influence of NIHL on daily life health conditions, 3) To explore the relationship between NIHL and workplace health problems, and 4) To compare the outcomes between workers who wear noise protection and those who do not. By addressing these research gaps and achieving these objectives, the paper aims to provide comprehensive insights into the impacts of NIHL on workers’ sleep, daily health conditions, and workplace well-being.

The structure of this paper is outlined as follows: The subsequent section provides an extensive review of the relevant literature. Section 3 presents the formulated hypotheses. In the following section, the research methodology is detailed, including the development of research instruments, data sampling, and data analysis techniques. Section 5 presents the empirical findings, which are further discussed in Section 6. The paper concludes with a discussion on the theoretical contributions, practical implications, limitations, and potential avenues for future research in Section 7.

## 2 Related work and hypothesis development

The literature on NIHL and its broader impacts on sleep quality, daily health conditions, and workplace well-being provides valuable insights into this research area. This section presents a comprehensive review of the relevant literature, highlighting key findings and identifying gaps that the current study aims to address.

Hearing protection is necessary to prevent NIHL, and many industries, especially those with high noise levels, have systematic NIHL prevention programs for their workers. However, in smaller workplaces or where there is a lack of organized health management and on-site monitoring, protection is often not worn. Hearing protection nonuse was most prevalent in the accommodation and food services (147 %), healthcare and social assistance (122 %), and educational services (128 %) industries, with mining workers at the highest risk of nonuse, and 65 % nonuse in food preparation and serving-related industries [8].

In a study examining the prevalence of noise exposure by industry and occupation, healthcare and social assistance had the highest risk of hypertension, shift workers were associated with hypertension and cardiovascular disease, and production workers in manufacturing had the highest risk of noise-induced hypertension [2]. Another occupationally exposed group is firefighters. They are subjected to hazardous noise levels from alarms and emergency response vehicles. In comparison to the recommended exposure limit of 85 dB(A) by the National Institute for Occupational Safety Health (NIOSH), the average firefighter was exposed to an average of 87.79 dB(A), and the average control group to 77.27 dB(A) [17]. The prevalence of occupational hearing loss is especially high among miners, and as mines look for ways to boost productivity, noise problems are getting worse. The NIOSH recommended exposure limit for noise is 85 dB(A), yet more than 80 % of South African platinum mine workers and 69 % of sand and gravel mine workers were exposed to noise levels over that threshold [18]. 96 % of miners in the western United States reported daily noise levels exceeding 90 dB(A) in a survey conducted there [19]. The average personal noise level was over 89.7 dB(A) according to a survey of three metal mining firms in China. In a study of noise exposure among factory workers, the mean was 81.3 dB(A) and the median was 82.29 dB(A). In the production of metal goods, cars, micro-businesses, collective enterprises, and private enterprises, individual noise exposure levels were relatively high [20]. Noise exposure has been determined to be between 65 and 90 dB(A) for workers in the semiconductor industry. Subjective weariness and irritation were noted by wafer production workers [21]. In various sectors of the automobile industry, employees working in units that performed roller and track trial testing, body assembly, engine manufacture, press forging, and engine manufacturing were subjected to excessive noise levels [22]. Textile manufacturing is another industry that has significant noise risks. Different work areas within textile mills have reported varying noise levels, including weaving (95–100 dB(A)), spinning (90–95 dB(A)), and packaging (70–80 dB(A)) [23].

The health impacts of noise exposure are influenced by a complex interplay of factors, including noise characteristics and the context of the environment [24]. Additionally, individual differences such as personality traits also play a significant role in how noise affects health, affecting sensitivity, annoyance, and loudness perception, as shown in a study by Abbasi, Etemadinezhad [25]. The health impacts, particularly those on sleep, are significant and are associated with life quality. Chronic partial sleep deprivation has negative consequences on sleep, including noticeable weariness, increased sluggish vigilance, and worse daily performance and general quality of life [26]. Noise, as a socio-psychological factor, activates the sympathetic nervous system and the hypothalamic-pituitary-adrenal axis, leading to hypertension [2,27]. It can increase levels of adrenaline, noradrenaline, and cortisol, in that order. Catecholamine levels, which are stress hormones, also increase significantly with increased noise exposure, leading to increased blood pressure and oxidative damage caused by constant noise exposure in the workplace. This can lead to other health problems associated with stress, including neurodegenerative diseases, reproductive function, kidney disease, and cancer [27]. Furthermore, heart rate significantly increased in response to stress, exercise, and different activities when examining the association between noise exposure and cardiovascular parameters [22]. According to studies [28,29], excessive noise at work can raise blood pressure, heart rate, and cardiac output through the release of stress hormones like catecholamines. Other studies also demonstrate that the type of noise has an impact on heart rate outcomes [30,31]. Lee, Chen [32] suggests that the accelerated process of inflammation, which connects atherosclerosis and arterial thrombosis, is a key factor in thrombus formation and has a negative impact on heart rate outcomes in addition to noise levels. It has been demonstrated that noise exposure at work is linked to high systolic and diastolic blood pressure as well as an increased risk of developing hypertension [33].

In summary, the existing literature provides a foundation for understanding the prevalence and audiological aspects of NIHL. However, there is a dearth of research investigating the specific impacts of NIHL on sleep quality, daily health conditions, and workplace well-being. Furthermore, limited research has explored the differences between workers who wear noise protection and those who do not. Understanding the differential impacts of NIHL based on the usage of protective measures is crucial for developing effective prevention strategies.

**Fig. 1:**
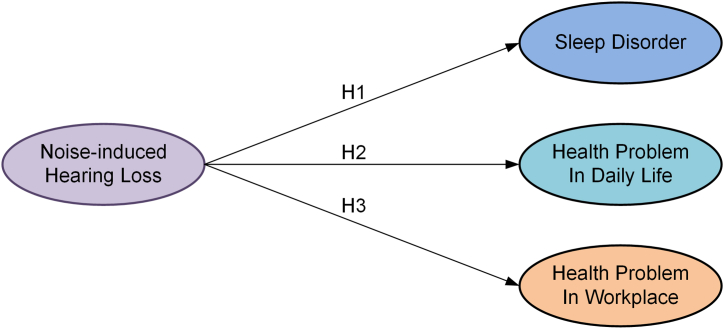
Research model.

Fig. 1 displays the research model. The research model for this study aims to investigate the impacts of NIHL on sleep quality, daily health conditions, and workplace well-being among workers. The model posits that NIHL, as an independent variable, will have a direct and significant effect on sleep disturbances, including sleep duration, efficiency, and the presence of sleep disorders. It also proposes that NIHL will be associated with daily life health conditions. Additionally, the model suggests that NIHL will impact well-being in workplace.

## 3 Research methodology

### 3.1 Instrument

The design of the survey tool for this investigation prioritized the precise evaluation of NIHL, sleep disorders, daily life health issues, and workplace health issues. We employed a mix of well-established scales and novel items to ensure a comprehensive appraisal of these variables.

The Hearing Handicap Inventory for Adults (HHIA) was utilized to measure NIHL. This tool assesses the social and emotional impacts of hearing loss (Newman et al., 1990). In this study, we tailored the HHIA to focus specifically on NIHL within a workplace context.

Sleep disorders were assessed with the General Sleep Disturbance Scale (GSDS) [34], which is rooted in the Pittsburgh Sleep Quality Index (PSQI) [35]. This well-validated instrument includes seven facets encompassing subjective sleep quality, sleep onset latency, sleep duration, habitual sleep efficiency, sleep disruptions, the use of sleep medication, and daytime impairment.

We developed a new scale to measure health issues in everyday life and the workplace, based on the framework established by Brazier, Jones [36]. To ensure the scientific integrity of the scale development process, the tool was designed collaboratively by the authors, three researchers in related fields, and four workers exposed to noisy environments. The questionnaire items were crafted to intuitively assess workers' health problems without deviating from established literature. After creating a draft version of the questionnaire, content validity was confirmed through feedback from subject matter experts. Exploratory factor analysis was then conducted to refine the items further. Finally, the finalized measurement tool was incorporated into the survey, and confirmatory factor analysis (CFA) was performed to validate the structure of the scale.

Table A1 provides an overview of the constructs and items utilized in our study's instruments, detailing the description of each item and its respective reference. All items were measured using a 5-point Likert scale. For the construct of noise-induced hearing loss, we employed three items based on Newman et al. (1990) to assess how hearing loss affects understanding coworkers, daily life, and communication with family. The sleep disorder construct, referenced from Choi et al. (2012), includes three items evaluating tiredness, poor sleep quality, and difficulty falling asleep within the last week. Health problems in daily life, referenced from Brazier et al. (1993), consist of three items addressing the inability to manage daily stress, enjoy daily life, and maintain confidence in living a normal life due to health issues. Similarly, health problems in the workplace, also based on Brazier et al. (1993), evaluate the impact of health issues on learning, remembering, concentration at work, workplace stress, and physical activity at work.

### 3.2 Data

This investigation's conceptual framework was validated via offline survey data collection. We aimed to enhance worker health and welfare via a survey disseminated by the Korean Confederation of Trade Unions to on-the-ground employees. Data collection spanned from July 8 to October 30, 2022. Collaborating with workplace health management networks, the investigators received necessary approvals from health management officials who administered the survey. The opening page of the survey explained its academic purpose and potential publication, ensuring informed consent from every participant. Only individuals who consented to publication took part in the survey. For culinary industry workers, hard-copy questionnaires were handed out directly to individuals who had been briefed about the purpose, methods, procedures, anonymity, and the right to withdraw at any stage without repercussions. It took roughly 10 min to complete the survey. The data collected was securely encrypted and stored on the personal computer of the researcher. After the completion of the study, it was disposed of appropriately. The survey was distributed to a total of 1500 participants. After the necessary deletions of incomplete or inconsistent responses, a total of 1285 valid questionnaires remained and were used in the analysis.

Table 1 presents the demographic characteristics of 1285 participants in the study. The gender distribution shows 431 males (33.5 %), 852 females (66.3 %), and 2 individuals (0.2 %) who did not respond. Age distribution spans from the 10s–60s, with the most represented group being those in their 50s at 42.9 %. Regarding industry, the majority of participants work in cooking (60.0 %), followed by manufacturing (26.5 %), and a small percentage in other sectors (13.5 %). The table also notes some participants who chose not to disclose their age (2.3 %).

**Table 1:** Demographic characteristics of the samples.

Demographics	Item	Subjects (*N* = 1285)	
		Frequency	Percentage
Gender	Male	431	33.5 %
	Female	852	66.3 %
	Not Respond	2	0.2 %
Age	10s	1	0.1 %
	20s	59	4.6 %
	30s	214	16.7 %
	40s	426	33.2 %
	50s	551	42.9 %
	60s	30	2.3 %
	Not Respond	4	0.3 %
Industry	Cooking	771	60.0 %
	Manufacturing	340	26.5 %
	Others	174	13.5 %

## 4 Analysis and results

In this research, we implemented the Partial Least Squares (PLS) technique to deal with formative factors and an extensive array of constructs. PLS is particularly effective for research involving intricate prediction models, especially those that contain multiple constructs, inclusive of formative constructs [37]. We utilized a two-stage process, as recommended by Hair, Anderson [38], for scrutinizing the measurement and structural models in terms of reliability, along with convergent and discriminant validity. This process is highly suitable for complex research models laden with a significant quantity of constructs.

### 4.1 Common method bias (CMB)

In research involving self-report measures, common method bias (CMB) poses a potential threat to the validity of the findings. CMB refers to spurious covariance shared among variables due to the method of data collection or the source of data, rather than the constructs the measures represent [39]. To address the potential for CMB in this study, several procedural and statistical remedies were employed. Procedurally, we ensured anonymity and confidentiality of the responses to reduce evaluation apprehension and social desirability bias. The instructions were clear and the questionnaire items were straightforward to avoid confusion and reduce respondent fatigue. Statistically, Harman's single-factor test was used as a post hoc analysis to check for the presence of CMB [40]. All items from the study were loaded into an exploratory factor analysis to see if a single factor emerged, or if one general factor accounted for the majority of the covariance among the measures. If such a factor were to exist, it would be an indication of CMB. Furthermore, the marker variable technique was employed [41]. A theoretically unrelated construct was included in the survey, and its correlation with the main constructs of the study was evaluated. If the correlations were high, it would suggest that CMB might be present. Lastly, we applied the PLS approach, as it is a well-suited method for dealing with potential method bias [37]. In summary, through these procedural and statistical techniques, the impact of common method bias on this study's findings has been minimized, thus increasing the confidence in the validity of the results.

### 4.2 Measurement model

A measurement model assesses the properties of the indicators used to measure constructs in the study. This includes the examination of the reliability and validity of the measures. In this study, the measurement model was evaluated using the two-step approach suggested by Hair, Anderson [38].

First, the reliability of the constructs was assessed. Internal consistency reliability, which indicates the degree to which items within each construct are correlated, was examined using Cronbach's alpha and Composite Reliability (CR). According to Nunnally [42], Cronbach's alpha values of 0.7 or above are considered acceptable. Next, the validity of the measures was evaluated. This includes convergent validity and discriminant validity. Convergent validity, the degree to which multiple items measure the same concept agree, was assessed through Average Variance Extracted (AVE) and factor loadings. According to Hair, Hollingsworth [43], AVE values of 0.5 or more and factor loadings of 0.6 or above suggest adequate convergent validity. Table 2 describes the reliability and validity of the scales.

**Table 2:** Reliability and validity.

Construct	Items	Mean	St. Dev.	Factor Loading	Cronbach's Alpha	CR	AVE
Noise-induced Hearing Loss	NIHL1	3.007	1.159	0.876	0.880	0.925	0.804
	NIHL2	3.554	1.103	0.911			
	NIHL3	3.529	1.122	0.903			
Sleep Disorder	SDO1	2.219	1.041	0.897	0.820	0.893	0.735
	SDO2	1.956	1.062	0.843			
	SDO3	2.475	1.012	0.832			
Health Problem in Daily Life	HPL1	3.014	1.104	0.824	0.806	0.886	0.721
	HPL2	2.947	1.174	0.889			
	HPL3	2.399	1.087	0.833			
Health Problem in Workplace	HPW1	2.451	1.028	0.851	0.688	0.831	0.625
	HPW2	3.883	0.842	0.647			
	HPW3	2.385	1.006	0.855			

Discriminant validity, the degree to which measures of different concepts are distinct, was evaluated through the Fornell-Larcker criterion and Heterotrait-Monotrait (HTMT). Fornell and Larcker [44] suggest that the AVE of each construct should be higher than its highest squared correlation with any other construct, which confirms discriminant validity (Table 3). The HTMT matrix is an approach that can be used for assessing discriminant validity. The HTMT ratio of correlations is a relative measure comparing the within-trait correlations (monotrait) against the between-trait correlations (heterotrait). Lower values of the HTMT ratio suggest greater discriminant validity [45]. In our study, the HTMT values were less than the commonly used threshold of 0.85 [46], suggesting that discriminant validity was achieved among the constructs (Table 4).

**Table 3:** Fornell-Larcker scale results.

Construct	1	2	3	4
1. NIHL	0.897			
2. Sleep Disorder	0.263	0.858		
3. Health Problems in Daily Life	0.296	0.356	0.849	
4. Health Problems in Workplaces	0.345	0.600	0.519	0.790

**Table 4:** HTMT matrix.

Construct	1	2	3	4
1. NIHL				
2. Sleep Disorder	0.297			
3. Health Problems in Daily Life	0.345	0.438		
4. Health Problems in Workplaces	0.436	0.794	0.699	

### 4.3 Hypothesis test

#### 4.3.1 Main effects

**Fig. 2:**
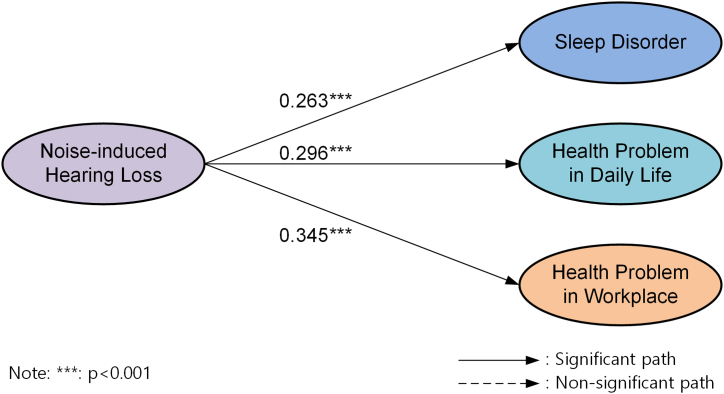
The path coefficients of the research model.

Structural Equation Modeling (SEM) was implemented via the PLS method to examine the proposed relationships among the constructs. We employed the bootstrap resampling technique (with 5000 resamples) to determine the significance of the path coefficients within the conceptual framework. The findings from this analysis are depicted in Fig. 2.

As anticipated, NIHL significantly and positively influences sleep disorder (*β* = 0.263, *t* = 9.957), health problems in daily life (*β* = 0.296, *t* = 10.793), and health problems in the workplace (*β* = 0.345, *t* = 13.814). This robustly endorses hypotheses H1, H2, and H3. Taken together, the proposed model accounts for approximately 6.9 percent of the variance in sleep disorders, 8.7 percent of the variance in health problems in daily life, and 11.9 percent of the variance in health problems encountered in the workplace. Table 5 describes the test results of structural model.

**Table 5:** SEM results.

H	Cause	Effect	Coefficient	*T*-value	*P*-value	Hypothesis
H1	NIHL	Sleep Disorder	0.263	9.957	<0.001	Supported
H2	NIHL	Health Problems in Daily Life	0.296	10.793	<0.001	Supported
H3	NIHL	Health Problems in Workplace	0.345	13.814	<0.001	Supported

#### 4.3.2 MGA

Table 6 displays the results from an MGA conducted to discern differences in the impacts of NIHL on Sleep Quality, Health Problems in Daily Life, and Health Problems in the Workplace, between two distinct groups: the Noise Protection Wearing Group and the Noise Protection Non-Wearing Group.

**Table 6:** MGA test between noise protection wearing group (A) and noise protection non-wearing group (B).

Cause	Effect	*β*_A_	*β*_*B*_	Difference (*β*_A_ - *β*_*B*_*)*	2-tailed *p-*value
NIHL	Sleep Disorder	0.271***	0.304***	−0.033	0.000
NIHL	Health Problems in Daily Life	0.226***	0.410***	−0.184	0.000
NIHL	Health Problems in Workplace	0.275***	0.465***	−0.190	0.000

Note: A indicates Noise Protection Wearing Group and B indicates Noise Protection Non-Wearing Group.

Specifically, for the impact of NIHL on sleep disorder, the path coefficient is greater by 0.033 in the Non-Wearing Group, suggesting a more adverse effect on sleep disorder in the absence of noise protection. Similarly, the impact of NIHL on health problems in daily life is greater by 0.184 in the non-wearing group, implying a more negative effect on daily life health problems when noise protection is not worn. For health problems in the workplace, the path coefficient is greater by 0.190 in the non-wearing group, demonstrating a more substantial impact of NIHL on workplace health problems when noise protection is not used.

In conclusion, these results underscore the importance of wearing noise protection in contexts with potential NIHL exposure, as it appears to alleviate the negative effects of NIHL on sleep quality, health problems in daily life, and health problems in the workplace.

## 5 Discussion

### 5.1 Main effects

The analysis conducted on the relationship between noise-induced hearing loss (NIHL) and various health outcomes provides substantial insight into the broader consequences of noise exposure in occupational settings.

The significant positive coefficient (0.263) and the highly significant *p-*value (<0.001) for the relationship between NIHL and sleep disorder supports the hypothesis that NIHL adversely affects sleep quality. These results align with previous research by Themann and Masterson [47], who found that exposure to high levels of noise disrupts normal sleep patterns, leading to increased sleep disturbances. The magnitude of the coefficient in this study suggests that as the severity of hearing loss increases, sleep disorders become more pronounced, corroborating findings by Johnson, Billings [48] which highlighted that chronic noise exposure is closely linked to poorer sleep quality. he influencing mechanism here could involve the disruption of circadian rhythms and the triggering of the stress response, which together exacerbate sleep disorders. This relationship underscores the physiological and psychological pathways through which noise impacts sleep, likely through increased stress and auditory stimulation that prevents the attainment of deep sleep.

The analysis shows a coefficient of 0.296 with a *p-*value of <0.001, indicating a strong positive effect of NIHL on health problems in daily life. This supports the hypothesis and is consistent with the literature that documents the extensive impact of auditory health on overall well-being [49]. This relationship might be mediated by the increased stress and cognitive fatigue associated with coping with hearing loss, which Astuti, Suhardi [50] suggested can exacerbate general health issues. The significant association between NIHL and health problems in daily life likely related to the cognitive overload and stress associated with trying to communicate in a noisy environment, which can lead to broader health deterioration. The finding underscores the need for comprehensive health monitoring and intervention strategies for individuals in noisy work environments.

With the highest coefficient of 0.345 and a *p-*value of <0.001, the results strongly support the hypothesis that NIHL significantly impacts health problems in the workplace. This finding is critical as it highlights the direct link between occupational noise exposure and workplace health, echoing the conclusions of Fried, Melamed [51] who reported similar associations in industrial settings. The result suggests that workers with NIHL may experience a range of health issues that can affect their productivity and safety at work, possibly due to the distraction and communication barriers imposed by hearing impairment. The potential mechanisms here could involve both the direct impact of noise on hearing capabilities and the indirect effects on stress levels, which can lead to a decrease in overall workplace efficiency and an increase in health-related absences. The comparative severity of this relationship suggests that workplace noise not only affects physical health but also extends to mental health challenges, a point that has been under-discussed in the literature but noted by Gritzka, MacIntyre [52] in a study on occupational health.

In summary, this study's findings elucidate the profound impacts of NIHL on sleep quality, daily health, and workplace well-being. Each of these relationships has been robustly supported by a strong statistical basis and aligns well with existing research, highlighting the multifaceted consequences of noise exposure. These insights emphasize the importance of effective hearing conservation programs and workplace health policies that could mitigate the adverse effects of noise exposure on workers' health. Future research should explore the specific mechanisms through which noise exposure exacerbates these health issues and test interventions that could alleviate the negative impacts identified in this study.

### 5.2 MGA

Our findings delineate the distinct influence of NIHL on sleep disorders, health problems in daily life, and health problems in the workplace among workers, and how the lack of noise protection amplifies these adverse impacts. These results provide further nuance to the current body of literature, underscoring the crucial role of noise protection in attenuating the detrimental effects of NIHL.

Our analysis demonstrates that NIHL has a more pronounced effect on sleep disorders in the Noise Protection Non-Wearing Group, with a higher path coefficient by 0.033. This finding corroborates prior studies suggesting that noise exposure disrupts sleep quality [24], and extends this understanding by illustrating how the lack of noise protection exacerbates this disruption. This suggests that inadequate noise protection may amplify the risk of sleep disorders among workers exposed to hazardous noise levels, primarily due to the intensifying effect of NIHL.

When considering health problems in daily life, our findings indicate a greater adverse effect in the Non-Wearing Group, with the path coefficient higher by 0.184. These results are in line with research by Basner, Babisch [13] that outlined the negative impacts of noise exposure on general health outcomes. This observation signifies that not utilizing noise protection could elevate the risk of developing health problems in daily life due to amplified NIHL.

For health problems in the workplace, our findings show a larger impact of NIHL in the Non-Wearing Group, with a higher path coefficient by 0.190. This finding complements existing studies [2,53] that reported adverse health effects related to workplace noise exposure. It further underscores the importance of noise protection measures in reducing the incidence of health problems in the workplace stemming from NIHL.

To summarize, these findings highlight the accentuating effect of not wearing noise protection on the impact of NIHL on workers' sleep quality, daily life health conditions, and workplace health problems. This emphasizes the crucial need for stringent adherence to noise protection measures in high noise exposure environments.

## 6 Conclusion

### 6.1 Theoretical implications

Our study has several theoretical contributions that advance the existing knowledge in the field of occupational health and safety, particularly regarding NIHL. To begin with, our research directly links NIHL to non-auditory impacts such as sleep disorders, health problems in daily life, and health problems in the workplace. This underscores the far-reaching implications of NIHL beyond merely auditory problems, something that has been less emphasized in prior studies.

Previous research has primarily focused on the auditory impacts of NIHL, such as hearing loss and tinnitus [54]. While important, this line of research has overlooked the potential ripple effects of NIHL on other facets of workers' health and well-being. Our study fills this gap by providing empirical evidence that NIHL can lead to sleep disorders and an array of health problems in daily life and the workplace. This expanded understanding of NIHL's impacts brings a more holistic perspective to the study of occupational noise exposure.

Another key contribution of our research is demonstrating the aggravating effect of not wearing noise protection on the impacts of NIHL. While previous studies have highlighted the importance of noise protection in mitigating auditory damage [7], our study takes a step further. We show that the absence of noise protection not only amplifies the risk of hearing loss but also exacerbates the impacts of NIHL on sleep quality and health problems. This novel finding underscores the need for research to look beyond the immediate auditory impacts of NIHL.

Furthermore, we have utilized a robust analytical approach – partial least squares method and multi-group analysis – that allows for a nuanced understanding of the relationships between our constructs. This contributes to the methodological development in this area of research and could set a precedent for future studies examining similar relationships.

Finally, the comprehensive nature of our study, incorporating diverse impacts of NIHL and the role of noise protection, provides a springboard for further research. Scholars could explore other potential non-auditory impacts of NIHL, investigate the efficacy of different types of noise protection, or examine the relationships we've identified in different populations or cultural contexts. In this way, our research not only expands current understanding but also points to new directions for further investigation.

### 6.2 Implications for practitioners

The findings of our study have important practical implications, particularly for occupational health and safety practitioners, employers, and policymakers. Foremost, understanding that NIHL can lead to sleep disorders and other health problems underscores the need to prioritize noise control measures in the workplace.

For organizations, particularly those in industries with high noise exposure like manufacturing, construction, and transportation, our findings should trigger a reevaluation of existing noise control strategies [55]. Active noise reduction technologies, such as sound barriers, noise damping, and vibration isolation, can be more extensively deployed. Passive noise control methods, such as providing employees with personal protective equipment like earplugs and earmuffs, should be considered essential [56]. Organizations can enhance workers' hearing protection and quality of life by forming institutional agreements with firms that customize and distribute user-specific earplugs, such as OTOS [58].

Our study shows the aggravating effect of not wearing noise protection, underlining the critical role of protective gear in mitigating the effects of NIHL. Employers should ensure the provision and proper use of noise protection equipment. This could involve training programs on the importance of wearing such equipment and the correct way to use it [59]. Additionally, organizational culture should encourage and normalize the use of such safety gear.

Health practitioners should also consider our findings in their approach to workplace health. Workers suffering from NIHL might be facing other health problems such as sleep disorders. These health concerns could be due to the psychological stress and physical discomfort that come with hearing loss [60]. Thus, a comprehensive health assessment and support system are needed to help workers cope with NIHL and its ripple effects.

Finally, policymakers should reflect these insights in occupational health and safety standards and regulations. Existing standards, such as the Occupational Safety and Health Administration's (OSHA) permissible noise exposure limits, could be reassessed in light of these findings [61]. Stricter noise exposure limits or more stringent requirements for noise control measures could be necessary to prevent NIHL and its attendant health problems.

### 6.3 Limitation and future research

Like all studies, the present one has limitations that provide avenues for future research. First, our study was cross-sectional, which limits the ability to make causal inferences about the relationships between NIHL, sleep disorders, and health problems. Future research could adopt a longitudinal design to clarify the direction of these relationships and examine how they develop over time. Second, our findings are based on self-reported measures, which can be subject to response bias. For example, individuals might underreport the severity of their hearing loss or overestimate their sleep quality. Future research might consider using objective measures, such as audiometric testing for hearing loss and polysomnography for sleep disorders. Thirdly, our study examined the impact of noise protection use but did not account for the quality and proper use of such equipment. Future studies could examine how the quality of hearing protection and the consistency of its use affect the relationship between noise exposure and health outcomes. Finally, while this study did not specifically investigate the impact of NIHL on appetite, future research could explore this aspect to understand the broader nutritional consequences of NIHL. Recognizing that NIHL may affect various physiological responses, it is essential to investigate how changes in appetite and eating behaviors could be systematically linked to occupational noise exposure.

## Ethics Statement

This study was approved by an institutional review board of a Catholic university. The IRB approval number of the Catholic university is MC22QISI0026. Informed consent was obtained in written form from all individual participants included in the study.

## Data availability

The data used in this study are available from the corresponding author upon reasonable request.

## CRediT authorship contribution statement

**Hyeon Jo:** Writing – original draft, Methodology, Formal analysis, Conceptualization. **Eun-Mi Baek:** Investigation, Data curation, Conceptualization.

## Declaration of generative AI and AI-assisted technologies in the writing process

During the preparation of this work the author(s) used ChatGPT in order to improve language and readability. After using this tool/service, the author(s) reviewed and edited the content as needed and take(s) full responsibility for the content of the publication.

## Declaration of competing interest

The authors declare that they have no known competing financial interests or personal relationships that could have appeared to influence the work reported in this paper.

## Figures and Tables

**Table A1:** List of Constructs and Items

Construct	Item	Description	Reference
Noise-induced hearing loss	NIHL1	I am afraid that if my hearing loss becomes severe, I will have to leave my current job.	Newman, Weinstein [62]
	NIHL2	I am worried that severe noise-induced hearing loss will affect my quality of life.	
	NIHL3	I am concerned that my family will be affected if my noise-induced hearing loss becomes severe.	
Sleep Disorder	SDO1	How worried are you about your current sleep problems?	Choi, Kim [34]
	SDO2	Do people around you believe your quality of life is suffering because of your sleep problems?	
	SDO3	To what extent do you think your sleep problems interfere with your daytime activities?	
Health Problem in Daily Life	HPL1	My health problems make it difficult for me to manage daily stress.	Brazier, Jones [36]
	HPL2	My health problem interferes with my ability to enjoy daily life.	
	HPL3	I am not confident about living a normal life because of my health problem.	
Health Problem in Workplace	HPW1	Does your health condition make it difficult for you to learn, remember, or concentrate in your current job?	Brazier, Jones [36]
	HPW2	Do you often feel stressed at work because of your health condition?	
	HPW3	Does your health condition make it difficult for you to be active in your current job?	
