# Supplementary material for: Impacts of noise-induced hearing loss on sleep, health, and workplace: Multi-group analysis
Source: Heliyon. 2024 May 8;10(9):e30861. doi: 10.1016/j.heliyon.2024.e30861 (PMC11107223; doi:10.1016/j.heliyon.2024.e30861)
Supplement: Multimedia component 1 [file mmc1.docx]

**Hearing Protection Use Questionnaire (For Employees)**

Greetings!

We are conducting a study to understand the current state of hearing protection usage. Our aim is to identify the prevalent practices and challenges associated with the use of sound protection equipment, such as earmuffs and earplugs, across different industries. This will help us develop better alternatives for hearing protection.

Please be assured that: Participation in this survey is voluntary. The information you provide will remain confidential and will only be used for research purposes. You can opt out of the survey at any point without any repercussions. We appreciate your time and hope you can complete this survey, regardless of your busy schedule.

Q1. Consent to Participate:

The information provided in this survey will remain confidential and is solely for research purposes. You have the freedom to decline participation at any time without any consequences.

Do you agree to participate?

☐ I agree

☐ I do not agree (If selected, exit survey)

Q2. Gender:

☐ Male

☐ Female

Q3. Age:

______ years

Q4. In which industry does your business operate?

☐ Manufacturing

☐ Construction

☐ Shipbuilding

☐ Cooking

☐ Other (please specify): ______

Please evaluate your ability to work if you've experienced a health issue in the past month and still came to work.

| Question | 1.  Strongly  Disagree | 2.  Disagree | 3.  Neither  Agree  Nor  Disagree | 4.  Agree | 5.  Strongly  Agree |
| --- | --- | --- | --- | --- | --- |
| I am afraid that if my hearing loss becomes severe, I will have to leave my current job. |  |  |  |  |  |
| I am worried that severe noise-induced hearing loss will affect my quality of life. |  |  |  |  |  |
| I am concerned that my family will be affected if my noise-induced hearing loss becomes severe. |  |  |  |  |  |
| How worried are you about your current sleep problems? |  |  |  |  |  |
| Do people around you believe your quality of life is suffering because of your sleep problems? |  |  |  |  |  |
| To what extent do you think your sleep problems interfere with your daytime activities? |  |  |  |  |  |
| My health problems make it difficult for me to manage daily stress. |  |  |  |  |  |
| My health problem interferes with my ability to enjoy daily life. |  |  |  |  |  |
| I am not confident about living a normal life because of my health problem. |  |  |  |  |  |
| Does your health condition make it difficult for you to learn, remember, or concentrate in your current job? |  |  |  |  |  |
| Do you often feel stressed at work because of your health condition? |  |  |  |  |  |
| Does your health condition make it difficult for you to be active in your current job? |  |  |  |  |  |

**Thank you for your response.**
